# Clinical Trial-Ready Patient Cohorts for Multiple System Atrophy: Coupling Biospecimen and iPSC Banking to Longitudinal Deep-Phenotyping

**DOI:** 10.1007/s12311-022-01471-8

**Published:** 2022-10-03

**Authors:** Alain Ndayisaba, Ariana T. Pitaro, Andrew S. Willett, Kristie A. Jones, Claudio Melo de Gusmao, Abby L. Olsen, Jisoo Kim, Eero Rissanen, Jared K. Woods, Sharan R. Srinivasan, Anna Nagy, Amanda Nagy, Merlyne Mesidor, Steven Cicero, Viharkumar Patel, Derek H. Oakley, Idil Tuncali, Katherine Taglieri-Noble, Emily C. Clark, Jordan Paulson, Richard C. Krolewski, Gary P. Ho, Albert Y. Hung, Anne-Marie Wills, Michael T. Hayes, Jason P. Macmore, Luigi Warren, Pamela G. Bower, Carol B. Langer, Lawrence R. Kellerman, Christopher W. Humphreys, Bonnie I. Glanz, Elodi J. Dielubanza, Matthew P. Frosch, Roy L. Freeman, Christopher H. Gibbons, Nadia Stefanova, Tanuja Chitnis, Howard L. Weiner, Clemens R. Scherzer, Sonja W. Scholz, Dana Vuzman, Laura M. Cox, Gregor Wenning, Jeremy D. Schmahmann, Anoopum S. Gupta, Peter Novak, Geoffrey S. Young, Mel B. Feany, Tarun Singhal, Vikram Khurana

**Affiliations:** 1 Department of Neurology, Building for Transformative Medicine Room 10016L, Brigham and Women’s Hospital and Harvard Medical School, 60 Fenwood Road, Boston, 02115 USA; 2 Division of Clinical Neurobiology, Department of Neurology, Medical University of Innsbruck, Anichstraße 35, 6020 Innsbruck, Austria; 3 Department of Radiology, Brigham and Women’s Hospital and Harvard Medical School, Boston, MA 02115 USA; 4 Department of Pathology, Brigham and Women’s Hospital and Harvard Medical School, Boston, MA 02115 USA; 5 Present Address: Department of Neurology, University of Michigan, Ann Arbor, MI 48103 USA; 6 Department of Pathology, Massachusetts General Hospital and Harvard Medical School, Boston, MA 02114 USA; 7 Department of Neurology, Massachusetts General Hospital and Harvard Medical School, Boston, MA 02114 USA; 8 Cellular Reprogramming, Inc., Pasadena, CA USA; 9 The Multiple System Atrophy Coalition, Inc., 7918 Jones Branch Drive, Suite 300, McLean, VA 22102 USA; 10 Department of Pulmonary, Sleep and Critical Care Medicine, Salem Hospital, MassGeneral Brigham, Salem, MA 01970 USA; 11 Department of Urology, Brigham and Women’s Hospital and Harvard Medical School, Boston, MA 02115 USA; 12 Department of Neurology, Beth Israel Deaconess Medical Center and Harvard Medical School, Boston, MA 02115 USA; 13 Laboratory of Neurogenetics, Disorders and Stroke, National Institute of Neurological, National Institute of Neurological Disorders and Stroke, Bethesda, MD 20892 USA; 14 Department of Neurology, Johns Hopkins University Medical Center, Baltimore, MD 21287 USA; 15 Department of Biomedical Informatics, Harvard Medical School, Boston, MA USA; 16 Division of Genetics, Department of Medicine, Brigham and Women’s Hospital, Boston, MA 02115 USA

**Keywords:** Multiple system atrophy, Stratification, Clinical trials, N-of-1 clinical trials, Induced pluripotent stem cells

## Abstract

Multiple system atrophy (MSA) is a fatal neurodegenerative disease of unknown etiology characterized by widespread aggregation of the protein alpha-synuclein in neurons and glia. Its orphan status, biological relationship to Parkinson’s disease (PD), and rapid progression have sparked interest in drug development. One significant obstacle to therapeutics is disease heterogeneity. Here, we share our process of developing a clinical trial-ready cohort of MSA patients (69 patients in 2 years) within an outpatient clinical setting, and recruiting 20 of these patients into a longitudinal “n-of-few” clinical trial paradigm. First, we deeply phenotype our patients with clinical scales (UMSARS, BARS, MoCA, NMSS, and UPSIT) and tests designed to establish early differential diagnosis (including volumetric MRI, FDG-PET, MIBG scan, polysomnography, genetic testing, autonomic function tests, skin biopsy) or disease activity (PBR06-TSPO). Second, we longitudinally collect biospecimens (blood, CSF, stool) and clinical, biometric, and imaging data to generate antecedent disease-progression scores. Third, in our Mass General Brigham SCiN study (*s*tem *c*ells *i*n *n*eurodegeneration), we generate induced pluripotent stem cell (iPSC) models from our patients, matched to biospecimens, including postmortem brain. We present 38 iPSC lines derived from MSA patients and relevant disease controls (spinocerebellar ataxia and PD, including alpha-synuclein triplication cases), 22 matched to whole-genome sequenced postmortem brain. iPSC models may facilitate matching patients to appropriate therapies, particularly in heterogeneous diseases for which patient-specific biology may elude animal models. We anticipate that deeply phenotyped and genotyped patient cohorts matched to cellular models will increase the likelihood of success in clinical trials for MSA.

## Introduction

Neurodegenerative synucleinopathies, including multiple system atrophy (MSA), are fatal, incurable diseases. MSA clinically presents with autonomic failure, parkinsonism (MSAp), ataxia (MSAc), and pyramidal tract dysfunction [1]. Differentiation from Lewy body diseases (Parkinson’s disease [PD] and dementia with Lewy bodies [DLB]) or late-onset ataxias can be challenging, especially early in the disease course. Neuropathologically, MSA is characterized by neuronal loss in multiple brain regions, widespread proteinaceous alpha-synuclein-rich inclusions in neurons and oligodendrocytes, and neuroinflammation [2–5]. Its etiology remains unclear. Familial forms of synucleinopathy caused by alpha-synuclein locus triplication [6, 7] or certain point mutations [8, 9] manifest with an MSA-like phenotype. Somatic copy number variations of the *SNCA* gene have also been associated with MSA [10].

Most clinical trials aimed at disease modification in neurodegenerative diseases like MSA have thus far failed [11–17]. A recent Global MSA Research Roadmap meeting was convened to establish barriers to therapeutics [18]. Important impediments include (i) difficulty to achieve early diagnosis; (ii) heterogeneity of the disease (a “one-size fits all strategy” may not work for therapy; (iii) inadequate biomarkers to track disease progression in a reasonable timeframe; (iv) animal models that fail to capture the complexity of the human disease.

The current sensitivity of MSA diagnosis is low [19–21]. This underscores the need for multimodal ancillary testing at the initial clinical workup for patients with a suspected parkinsonism-ataxia spectrum disorder or for patients that present with prodromal features, including REM sleep behavior disorder [22, 23]. Another prioritized recommendation of the Roadmap meeting included the development of clinical, imaging, and other biofluid markers that reliably track disease progression since clinical indices alone are poorly reflective of progression rate [24]. Ancillary testing includes autonomic function tests [25, 26], olfactory testing [27], 123I-MIBG cardiac scintigraphy [28], multimodal MRI assessment [29–31] (see also Kim et al. [32]), and positron emission tomography (PET) imaging of the translocator protein (TSPO) [33].

Emerging molecular diagnostic tests warrant mention. Skin biopsy for α-synuclein immunohistochemistry and assessment of nerve fiber density is now commercialized based on high sensitivity and specificity to distinguish synucleinopathy from other proteinopathies [34], with alpha-synuclein deposition predominating in somatosensory versus sympathetic adrenergic fibers in MSA versus PD, respectively [35, 36]. Seed amplification assays (SAA) including real-time quaking-induced conversion (RT-QuiC) or protein misfolding cyclic amplification (PMCA) of amyloidogenic alpha-synuclein conformers from cerebrospinal fluid [37] may distinguish PD from MSA and have also recently been commercialized [38]. Neurofilament light chain is increased in MSA compared to PD both in CSF and blood in early-stage disease [39, 40]. Finally, a recently developed radiotracer for alpha-synuclein detection in the brain [41] has reportedly shown promise for MSA in the clinic [42].

Because the complex biology of MSA may not be fully captured in animal models, a Roadmap recommendation was also made to establish induced pluripotent stem cell (iPSC)–based models of MSA. iPSCs are pluripotent human embryonic stem cell–like cells that are derived through somatic cell reprogramming (for example, of blood or fibroblast cells) and can thereafter be differentiated into any somatic tissue, including the central nervous system [43, 44]. iPSC technology enables a personalized model that can be generated from an individual MSA patient and can theoretically capture the genetic heterogeneity of the disease “in the dish” [45, 46].

**Fig. 1:**
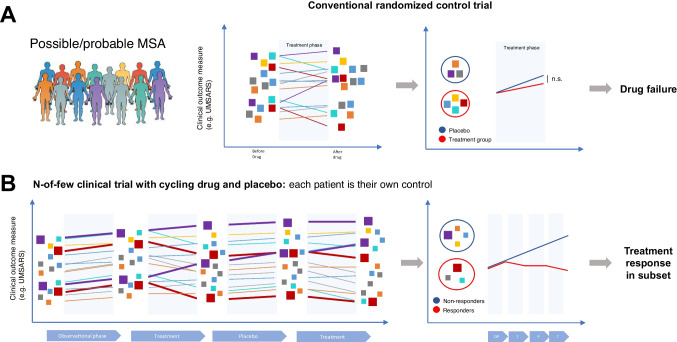
Longitudinal trial design may improve feasibility and interpretability for complex diseases like MSA. **A** A conventional randomized therapeutic trial begins with a biologically and clinically heterogenous patient population, indicated by schematic persons of many colors (left). Each patient has possible/probable MSA, depending on inclusion criteria, and is assigned randomly into a treatment vs. placebo arm, monitored clinically for the treatment duration. Outcome measurements are compared at the trial conclusion between the two groups. Two levels of heterogeneity may impede these trials. First, MSA patients may be too biologically heterogeneous: a subtype of true responders may be missed, the signal buried in noise. Second, patients may also be too clinically heterogeneous: noisy clinical outcome measures may mean a patient population large enough to track is not feasible, especially for drugs with modest effect size. **B** In a complementary longitudinal paradigm, a thoroughly investigated patient population enters an observational phase in which deep clinical, biomarker and imaging phenotyping is carried out. In this paradigm, each patient is their own control: phenotypic progression in a “treatment” phase is compared to an antecedent “observational” phase. In a “cycling” paradigm, alternating treatment and placebo windows are employed to ascertain true target engagement and efficacy signals. In the figure, the magenta patients are examples of non-responders, and the red patients are responders

These considerations suggest a strategic re-thinking of clinical-trial paradigms for MSA. A longitudinal paradigm might have merit, especially when coupled to biological stratification. In conventional cross-sectional randomized control trials, a cross-section of patients is randomly assigned to a drug or placebo, and success or failure depends on meeting some pre-defined endpoint (Fig. 1A). In this paradigm, biological heterogeneity in patients means that success depends on either a very strong drug effect or larger patient numbers [47]. In a longitudinal paradigm, a smaller group of deeply phenotyped patients is tracked according to a subset of these phenotypes, and then given a drug. The alteration of each patient’s phenotypic trajectory is then observed in response to a drug (Fig. 1B), thus each patient serves as their own control. This methodology is relatively new and untested and has pitfalls [48]. In order to verify true treatment effects, repeated “cycling “ of observations across multiple treatment and control periods may be necessary, but this is not easily achievable for a chronic progressive disease like MSA.

Here, we demonstrate the initial outcomes of a strategy for clinical trial recruitment from an outpatient setting. The strategy was geared to (i) biologically stratify MSA patients, and (ii) develop a “clinical trial-ready” patient population for longitudinal clinical trials. We report successful recruitment from our clinic population into a therapeutic interventional trial. In the process, we established 38 iPSC lines from MSA and related disorders, including 22 lines matched to the patient brain. These iPSC lines are now available to the research community. Propagating this approach in collaboration with other academic and industry partners may help overcome the impasses in therapeutic development for MSA and related disorders.

## Methods

### Polysomnography

Polysomnography entails the all-night recording of multiple inputs including limited electroencephalogram (EEG), electrooculogram (EOG), chin electromyogram (EMG), leg EMG, airflow signals, respiratory effort signals, oxygen saturation, body position, electrocardiogram (ECG), and synchronized PSG video. REM without atonia is identified by excessive sustained muscle activity (tonic activity) during REM sleep, excessive transient muscle activity (phasic activity) during REM sleep, or any chin EMG activity at least twice the amplitude of the REM atonia during REM sleep. Tonic activity is defined as an epoch of REM sleep exhibiting a chin EMG amplitude at least two times greater than the REM atonia tone or the lowest tone during NREM sleep if REM stage atonia is not present. Phasic activity is defined as at least 50% of 3-s “mini epochs” within the 30-s epoch containing bursts of transient EMG activity in the chin or limb leads. The transient muscle activity must be 0.5–5 s with an EMG amplitude at least two times greater than the REM atonia tone or the lowest tone during NREM sleep if REM stage atonia is not present (https://aasm.org/clinical-resources/scoring-manual/).

### Autonomic Testing

We evaluate MSA patients for the presence of dysautonomia using surveys and objective testing [49]. The BWH protocol evaluates all main autonomic branches, small skin fibers, cerebral blood flow, which is frequently affected in MSA, and respiratory failure using objective testing. Autonomic testing includes deep breathing (evaluates cardiovagal parasympathetic system), Valsalva maneuver (evaluates sympathetic adrenergic system), tilt test (evaluates sympathetic and parasympathetic system), sweat test (evaluates postganglionic sudomotor system), and evaluation of plasma norepinephrine levels in supine and standing position. Electrocardiogram, continuous and intermittent blood pressure, end-tidal CO_2_, finger pulse oximetry, and cerebral blood flow velocity (CBFv) in the middle cerebral artery (MCA) using transcranial Doppler are acquired and processed during autonomic testing. Results are graded using the Quantitative Scale for Grading of Cardiovascular Autonomic Reflex Tests and Small Fibers from Skin Biopsies (QASAT) [49].

### Urodynamics

Urodynamic testing for neurogenic lower urinary tract dysfunction includes postvoid residual urine measurement (PVR) to identify incomplete emptying (via ultrasound or via straight catheterization), uroflowmetry to measure urine flow rate over time, complex cystometrography to measure changes in intravesicular pressure related to change in volume during filling, pressure flow analysis to measure the relationship between detrusor pressure and urine flow rate during voiding, and electromyography to assess the electric activity of the striated sphincteric muscles of the perineum and is performed during filling and voiding. Urodynamics with fluoroscopy (i.e., video urodynamics) facilitates visualization of the bladder and urethra during events of filling and emptying [50].

### Magnetic Resonance Imaging

Magnetic resonance imaging (MRI) at BWH is performed on a Siemens 3 T MAGNETOM Prisma MR system (Med System US Inc., Malvern, PA USA) using a standard research protocol, which includes MRI compatible with 3-D segmenting software (e.g., 3D MPRAGE T1WI sequence). In addition, we obtain DTI and DWI sequences. Volumetric measurement utilizes Freesurfer v6.0 running on a high-performance research computing cluster. We apply the Freesurfer Brainstem Substructures module to automate the segmentation of the pons, midbrain, medulla, and SCP. Each segmented MRI has been examined for accuracy by a radiologist and selected images have been reviewed by a senior radiologist on Freeview, a tool developed by Freesurfer developers to visualize Freesurfer output. For patients with > 1 MRI, Freesurfer’s longitudinal stream is being applied for intrasubject MRI analysis to increase precision.

### Positron Emission Tomography

We use [F-18]-fluorodeoxyglucose positron emission tomography (PET) (FDG)-PET to visualize characteristic patterns of hypometabolism in MSA including basal ganglia, pons, cerebellum, and more recently several neocortical regions [51–54]. In addition, we are using [F-18]PBR06, a second-generation TSPO-PET ligand with a longer half-life and high specific binding for TSPO, an 18 kilodalton-translocator protein (TSPO) expressed in microglia and astrocytes [33, 55, 56], for in vivo longitudinal tracking of neuroinflammatory changes in MSA patients and to perform pharmacodynamic measurements [57].

### CLIMB (Comprehensive Longitudinal Investigations of Multiple Sclerosis at the Brigham and Women’s Hospital)

The primary goal of the biorepository is to prospectively collect blood and stool samples from individuals with multiple sclerosis (MS) at different stages of the disease and while on different disease-modifying therapies, to compare them to samples collected from neurologic disease controls (including MSA), autoimmune controls, and healthy controls. Serum, plasma, and peripheral blood mononuclear cells are isolated from blood samples and cryopreserved. Immortalized lymphoblastoid cell lines, tumor cell lines, pluripotent stem cells, and derived cells can be generated. Routine downstream analyses include immune profiling (serum autoantibody signatures, transcriptional profiling of immune-cell subsets, functional analysis of immune cell activation, differentiation, polarization, and cytokine production). Whole-stool samples are collected without chemical preservatives and frozen at − 80 °C. Samples are later aliquoted into 1-mL vials and used for 16S rRNA sequencing, cultivation of strains of bacteria from the microbiome, or transfer into animal models of disease.

### HBS (Harvard Biomarker Study)

The Harvard Biomarker Study is a Harvard-wide, longitudinal, case–control study of over 3300 participants with PD, memory impairment, and controls (as of October 2021). High-quality biospecimens and high-resolution clinical phenotypes are tracked at four to five visits over a 5-year period. To minimize bias, age- and gender-similar healthy controls are enrolled from the source population, and case and control samples are processed in parallel and quality-controlled. Biospecimens tracked include cerebrospinal fluid (CSF), plasma, serum, buffy coat, whole blood, cryopreserved white blood cells, microRNA, PAXgene RNA, DNA, immortalized human lymphoblastoid cell lines, and, ultimately, brain autopsy. Hematological variables (e.g., complete blood counts) and sample processing steps are monitored. Skin biopsies for the generation of fibroblasts and iPSCs are performed on a selected subset. Gold-standard movement disorder motor scales are performed at each longitudinal in-person visit, including the Unified MSA Rating Scale (UMSARS) and Brief Ataxia Rating Scale (BARS) for MSA patients. Detailed longitudinal clinical phenotypes include demographics, ancestry, blood pressure, BMI, medication history, environmental exposures (e.g., PD-relevant detailed smoking, caffeine, pesticide, herbicide, etc. exposures), family history, and social history. Neuropsychologic/memory status is longitudinally monitored through the Mini Mental State Exam (MMSE), Geriatric Depression Scale, Queen Square Visual Hallucinations Inventory, and the Montreal Cognitive Assessment battery (the latter for the 60-month follow-up visit only). Sleep is longitudinally assessed by the REM Behavior Disorder Scale. Quality of life is assessed through the PDQ39 and SF-12 instruments. Downstream analyses in HBS include genomic, transcriptomic, metabolomic, and CSF profiling.

### Genetics Evaluation—Movement Disorder Protocol

Patients with a suspected genetic etiology, typically based on the young age of onset (< 50 years old) or a family history of at least one additional individual with a history of MSA or a related condition, are referred to the Neurogenetics clinic. At these appointments, patients receive extensive genetic counseling and have a brief physical exam and a three-generation family history is taken. Initial testing involves screening for genetic causes of Parkinson’s disease and/or ataxia, such as repeat expansion disorders that can mimic MSA [58] and copy number variants in the SNCA gene [7]. In addition, a next-generation sequencing panel of genes related to Parkinson’s disease and/or parkinsonism can be ordered, as well as specific assays to detect pathological expansions in genes of interest such as ATXN2 (SCA2) and ATXN3 (SCA3). If negative, whole-exome sequencing (WES) is considered next. Notably, we consider WES and whole-genome sequencing (WGS) only if there is still strong suspicion of a genetic cause after all other testing has been uninformative as there can be a considerable cost involved. Finally, we consider research re-analysis of the clinical sequencing data on select patients in which clinical reports are uninformative. The purpose of this research re-analysis is to discover novel genes and/or variants that did not meet the clinical lab’s threshold for reporting but may eventually be informative through additional translational research efforts.

### Whole-Genome Sequencing, Alignment, Variant Calling (from postmortem brain)

We extract genomic DNA from frozen brain tissue and quantify using the Quant-iT PicoGreen dsDNA Assay Kit (Thermo Fisher Scientific). PCR-free, paired-end, non-indexed libraries are generated using the TruSeq Library Preparation Kit (Illumina) according to the manufacturer’s protocols. Sequencing is performed on an Illumina HiSeq X Ten platform (v.2.5 chemistry) with 150 bp, paired-end cycles, applying a single library to each lane of a flow cell.

Following genome-sequencing, the raw sequence reads in a FASTQ file format are processed following the pipeline standard developed by the Centers for Common Disease Genomics (CCDG; https://www.genome.gov/27563570/). The sequence data are aligned to the human reference genome (hg38) using the Broad Institute’s implementation of the functional equivalence standardized pipeline. This pipeline incorporates the GATK Best Practices. Single-nucleotide variant and insertion-deletions are called using another Broad Institute workflow for joint discovery and Variant Quality Score Recalibration (VQSR). The Broad Institute workflows for sample processing and joint variant discovery are publicly available at https://githubcom/gatk-workflows/broad-prod-wgs-germline-snps-indels. The average sequence read depth per genome is 35X.

### Biometrics

We enroll individuals to participate in a quantitative behavioral phenotyping [59], in press) protocol that includes both in-person and remote assessments of motor and cognitive function. In-person quantitative assessments are conducted every 6 months in a “neurobooth,” consisting of consumer and laboratory grade cameras, eye trackers, microphones, and wearable sensors that capture behavioral data as individuals perform oculomotor, speech, limb motor, balance, and cognitive tasks. At-home assessments include two main components, a wrist sensor worn continuously for 1 week at 3-month intervals [60], and a weekly web-based computer mouse task, which involves clicking on targets as they appear in sequence on the screen [61]. This protocol enables us to capture quantitative and comprehensive phenotypic data from participants in natural and structured contexts in a relatively low burden manner.

### Skin Biopsy Protocol (for α-Synuclein Immunohistochemistry and Assessment of Nerve Fiber Density)

We collect three 3-mm skin punch samples at the following sites from both patients: 10 cm above the lateral malleolus, 10 cm above the lateral knee, and 3 cm lateral to the C7 vertebral prominence [34]. Briefly, tissues are fixed in Zamboni’s fixative for 24 h and frozen and cut into 50 μm sections. Tissue sections are stained with immunofluorescent antibodies against protein gene product (PGP9.5) and phosphorylated α-synuclein (clone #64, Wako Chemicals) using previously published protocols. A total of six tissue sections for each of the three biopsies is analyzed (18 tissue sections reviewed per patient). An image series of optical sections is acquired with a fluorescent microscope, with areas of interest imaged by confocal microscopy, at 2-μm intervals throughout the depth of the 50-μm section as a z-stack. Only regions where phosphorylated α-synuclein is completely co-localized within nerve fibers stained with PGP9.5 are considered positive as previously described [62].

### Brain Banking Protocol

Following removal, the brain is divided into the mid-sagittal plane. Half of the brain is fixed for 2 weeks in formalin for standard neuropathological examination (Figs. 5 and 6A) and the other half frozen at − 80 °C. Prior to freezing, the brainstem is separated from the cerebrum at the level of the midbrain. The cerebrum is sectioned coronally in approximately 1-cm-thick slices. The cerebellum is removed from the brainstem by sectioning through the cerebellar peduncles. The brainstem is then sectioned in the axial plane in approximately 0.5-cm-thick slices. The cerebellum is sectioned radially by starting centrally at the vermis and moving laterally. All of the sections are then frozen rapidly in liquid nitrogen vapor and placed into a − 80 °C freezer in plastic freezer bags (6 × 4 inch and 15 × 12 inch Minigrip clear zipper bags; Fisher Scientific, catalog numbers 22310023 and 22310029).

### Fibroblast Culture

At BWH, to generate fibroblasts, we first wash the skin biopsy several times in sterile phosphate-buffered saline and mince it into fine pieces upon removal of visible adipose tissue. In a polystyrene plate, a fine grid is cut into the bottom of each well, where small tissue pieces will loosely attach to intersecting molds, facilitating fibroblasts to grow out into the plate. Twenty-five-millimeter coverslips are gently placed on top and an additional medium is added to each well. Fibroblast culture medium consists of DMEM, 15% fetal bovine serum, GlutaMAX™ Supplement (1 ×), MEM Non-Essential Amino Acids Solution (1 ×), and Penicillin–Streptomycin (1 ×) (Gibco, ThermoFisher, Waltham, MA).

After 5 days, the medium is carefully removed using different stripettes for each well/dish and collected for mycoplasma testing. Whole medium changes are performed every 3 days thereafter, and following expansion, cells are frozen down for storage and reprogramming. A similar protocol is used at MGH, but initial plating is in T75 culture flasks and 10% fetal bovine serum is used.

### iPSC Generation

Human fibroblasts are subjected to mRNA reprogramming [63–65] using a cocktail incorporating engineered, chimeric transcription factors to accelerate lineage conversion. The resulting iPSC colonies were stabilized and expanded under xeno-free conditions by Cellular Reprogramming, Inc. (Pasadena, CA). Colonies are bulk-passaged from the most productive well to establish passage 1 iPSC cultures on rLaminin-521 (BioLamina, Sundbyberg, Sweden) in Nutristem XF media (Biological Industries, Kibbutz Beit-Haemek, Israel) and expanded in the same culture system until at least passage 3 before being frozen down for storage. Our iPSC lines are monitored biweekly for mycoplasma infection when in culture and checked for mycoplasma prior to cryopreservation. G-band karyotyping is performed prior to use for downstream experimentation.

The Partners Institutional Review Board (IRB) approved of study activities herein. IRB and FDA IND (investigational new drug) numbers: Harvard Biomarker Study (2003P000541), CLIMB (2017P001169), Fibroblast protocol (2009P000775), Neurobooth (2021P000257), Wrist sensor (2019P002752), experimental PET ligand (2016: 2016P002373, 2020: 2020P003415).

## Results

### P + A + MSA Clinical Cohort

**Fig. 2:**
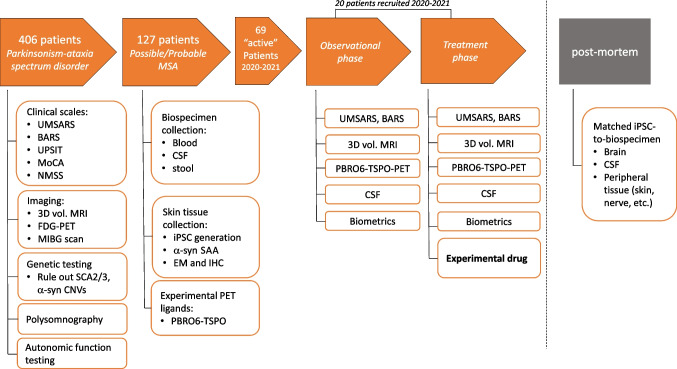
Detailed workflow for patients with potential MSA from initial presentation with parkinsonism-ataxia spectrum disorder to clinical MSA trial in the multidisciplinary P + A + MSA clinic. *Upper orange arrows:* from 406 patients in our database, 127 patients had suspected (possible or probable by diagnostic criteria) MSA. In the last 2 years, 69 of these patients have been tracked clinically and, of these, 20 patients enrolled in an investigator-initiated clinical trial that began with an observational phase followed by a treatment phase. *Lower:* (left) our current diagnostic workup. (Right) Testing that occurs in observational and treatment phases of our proposed longitudinal clinical trial paradigm. Abbreviations: UMSARS: Unified MSA Rating Scale; BARS: Brief Ataxia Rating Scale; UPSIT: University of Pennsylvania Smell Identification Test; MoCA: Montreal Cognitive Assessment; 3D vol. MRI: 3D volumetric magnetic resonance imaging; FDG-PET: fluoro-deoxy glucose positron emission tomography; MIBG: meta-iodobenzylguanidine; SCA: spinocerebellar ataxia; SAA: seed amplification assay; a-syn CNVs: alpha-synuclein copy number variations; CSF: cerebrospinal fluid; iPSC: induced pluripotent stem cell; EM: electron microscopy; IHC: immunohistochemistry; TSPO: translocator protein

The multidisciplinary P + A + MSA clinic for patients with parkinsonism plus, ataxia, and multiple system atrophy was established at Brigham and Women’s Hospital, a principal teaching affiliate of Harvard Medical School, in 2016. The clinic comprises two attending physicians, a movement disorders fellow-in-training, a neurogenetics fellow-in-training, a nurse practitioner, genetics counselor, physical therapist, and social worker. The aim is not only to provide thorough multidisciplinary care to patients, but to triage patients from the Mass General Brigham clinical catchment area (New England, USA, and beyond) for innovative clinical intervention trials. Over a period of 5 years, of the 406 patients with parkinsonism-ataxia spectrum disorders considered from Mass General Brigham, we classified 127 with MSA (Fig. 2, upper) at differing levels of certainty based on established clinical consensus criteria that rely on the clinical history and examination [19]. Of the 127 patients, 71 patients were alive as of January 2022 with a diagnosis of possible MSA (18/127) or probable MSA (53/127). Within the preceding 5-year period, 51 patients had died, 30 of which were neuropathologically confirmed for MSA, while 20 patients (19 patients diagnosed with probable MSA, 1 possible MSA case at last visit) did not undergo post-mortem examination upon death. Of note, one patient with suspected MSAc did not show any neuropathological signs of MSA at autopsy, and 5 patients classified with probable MSA at their last visit were lost to follow-up.

### Advanced Diagnostic Testing

As noted in the “Introduction” section, MSA is a disorder characterized by a progressive movement disorder and autonomic dysfunction. The motor dysfunction can be predominantly cerebellar ataxia (MSAc), parkinsonism (MSAp), or a mix of the two. Neuropathologically, the former correlates with olivopontocerebellar atrophy and the latter with striatonigral degeneration. Of the autonomic features, urinary dysfunction is most prevalent, but bowel, blood pressure control, respiratory function, and sexual function are frequently affected. This is most characteristically associated with the widespread loss of central autonomic neurons and preganglionic neurons in the paraspinal ganglia.

As noted above, our initial evaluation of potential MSA patients utilizes a panel of tests that enables a deeper investigation of these clinical patterns. For instance, urodynamic studies delineate the functional pathophysiology of urinary dysfunction and motivate appropriate treatments; fluoro-deoxy glucose PET (FDG-PET) and dopamine transporter SPECT scanning (“DaT scan”) provide a functional correlate of neuropathology and may be more sensitive than clinical examination to detect cerebellar and striatal dysfunction [66] or nigrostriatal dysfunction, respectively. Structural MRI is standard and readily available. There are classical findings that suggest MSA in the appropriate clinical settings, but these are variably present and often late-stage findings [67]. In contrast, 3D volumetric MRI, in conjunction with statistical and computational methods, offers a very promising approach to early differential diagnosis by identifying combinations of brain regions that are most likely to degenerate in atypical parkinsonism-ataxia spectrum disorders [30, 31] (also see accompanying submission, Kim et al. [32]). We assess 2D and 3D MRI routinely in all of our suspected MSA patients.

Additional testing can further refine the diagnosis, but not every test will be performed on each patient. Some of them, like radiotracer studies, are not without their risk and should be judiciously requested. Table 1 lists 10 tests we consider to have the most discriminatory potential based on the cited literature. We matched these tests to four commonly encountered clinical scenarios in our clinic (see “Methods” for more details). Each of these tests can be performed within the clinic or as a clinically approved and reportable diagnostic test. To date, we have utilized seed amplification of amyloidogenic alpha-synuclein from cerebrospinal fluid in the research context, but standardization and commercialization of this test should enable it to be routinely used for diagnostic purposes in the near future [38].

**Table 1:** Rational and selective testing according to clinical presentation facilitates early differential diagnosis

A					
		Clinical presentation			
		Parkinsonism + OH	Ataxia + UD	Ataxia—parkinsonism	Suspected familial ataxia-parkinsonism*
Selective investigations	MoCA	x			
	UPSIT	x			
	FDG-PET	x	x	x	
	DaT SPECT		x	x	
	MIBG	x			
	Skin: pa-syn	x	x		
	Skin: nerve fiber density	x			
	QSART	x			
	Polysomnography	x	x	x	
	Genetic testing	x	x	x	x
Differential diagnosis		PD, DLB, MSA, SCA2/3	MSA, SCA2/3, PSP	MSA, SCA2/3, PSP	MSA, SCA2/3, other genetic disorder*
B					
	*n*	%		*n*	%
Active set**	69	100	Polysomnography	12	17.39
UMSARS	69	100	Genetic testing	10	14.49
3D vol. MRI	52	75.36	UPSIT	7	10.14
FDG-PET	23	33.33	Skin Biopsy	7	10.14
Autonomic testing	19	27.54	DaT SPECT	5	7.25
MoCA	14	20.29	Lumbar puncture	4	5.80
Urodynamics	12	17.39	MIBG	3	4.35

A. Four clinical phenotypes/scenarios: (i) in a patient presenting with parkinsonism and autonomic dysfunction, the presence of peripheral neuropathy on skin biopsy and an abnormal MIBG scan indicating peripheral sympathetic cardiac denervation [68] is more consistent with Lewy body disease than MSA [69, 70]. Autonomic function tests in conjunction with QSART help in localizing the autonomic pathology (see “Methods”; [71]). Additionally, anosmia is more likely in a PD patient than in MSA or PSP [72] and is detected via UPSIT assessment. REM sleep behavior disorder on polysomnography in such a patient indicates a synucleinopathy is most likely [73]. Cognitive dysfunction, particularly in the visuospatial and recall domains, would be more suggestive of Lewy body disease than MSA [74]. (ii) In a patient with ataxia and urinary dysfunction, an FDG-PET and DaT scan indicating striatonigral degeneration, in conjunction with a skin biopsy for immunohistochemical detection of alpha-synuclein, can reliably pinpoint the diagnosis as a synucleinopathy [75, 76]. (iii) Similarly, in a patient with ataxia but without parkinsonism, a positive DaT scan and FDG-PET demonstrating striatal dysfunction would increase suspicion for MSA. (iv) In a patient with ataxia or parkinsonism, either with early onset (< 50yo) or with one or more first-order relatives with neurodegenerative diagnosis, genetic testing is performed to rule out common mimics, particularly SCA2, SCA3, and synuclein copy-number variants [77]. With negative testing, we proceed to repeat-expansion disorder profiling, whole-exome, and whole-genome sequencing, initially through clinical vendors and eventually to research (Harvard Medical School and Brigham and Women’s Hospital Clinical Genome Analysis Platform; CGAP)

B. Numbers of tests performed in our active patient cohort

Abbreviations: *OH*, orthostatic hypotenstion; *UD*, urinary dysfunction; *MoCA*, Montreal Cognitive Assessment; *UPSIT*, University of Pennsylvania Smell Identification Test; *FDG-PET*, fluoro-deoxy glucose positron emission tomography; *DaT SPECT*, dopamine transporter single-photon emission computed tomography; *MIBG*, meta-iodobenzylguanidine; *pα-syn*, phosphorylated a-synuclein; *QSART*, quantitative sudomotor axon reflex test; *PD*, Parkinson’s disease; *DLB*, dementia with Lewy bodies; *MSA*, multiple system atrophy; *SCA*, spinocerebellar ataxia; *PSP*, progressive supranuclear palsy; *UMSARS*, Unified MSA Rating Scale

^*^More recently, several cases of multiplex families of unknown genetic cause have been described, where post-mortem analysis confirmed diagnosis of MSA in some family members; therefore, genetic testing is included here in the context of MSA upon exclusion of common MSA mimics (Multiple-System Atrophy Research Collaboration NEJM 2013) [78]

^**^Last visit 2019 or later

**Fig. 3:**
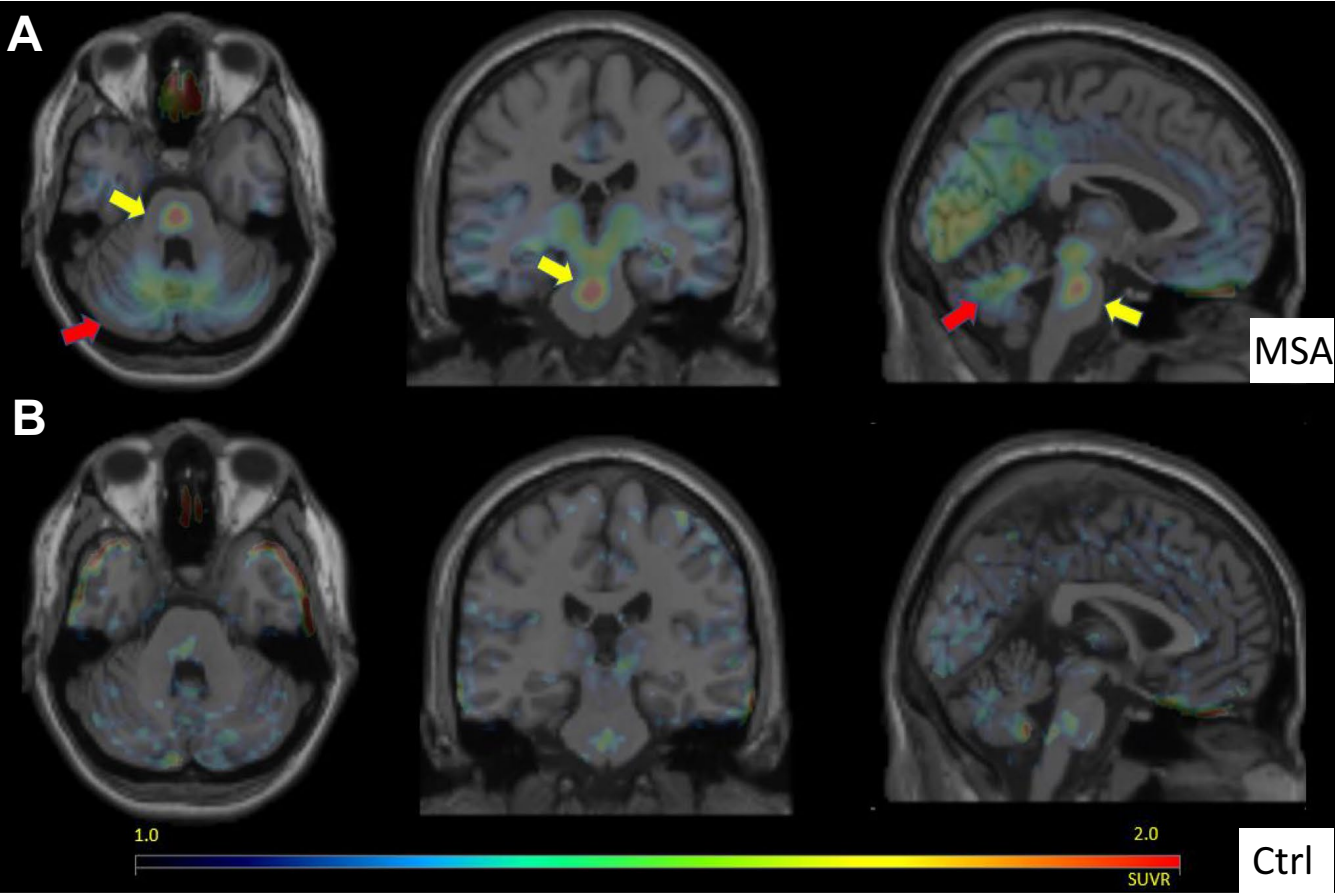
[F18]PBR06-TSPO-PET for Translocator Protein as a surrogate for CNS neuroinflammation. **A** Markedly increased radiotracer accumulation in basis points (yellow arrows) and cerebellar white matter (red arrows) in an MSA-C patient (top row). **B** A healthy control comparison

**Fig. 4:**
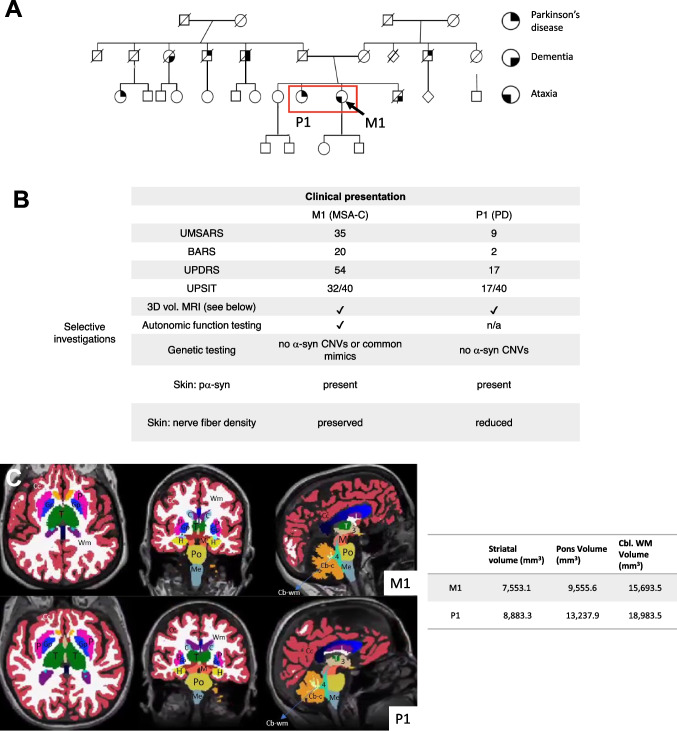
A multiplex family involving cases with MSA, Parkinson’s disease, and dementia. **A** A three-generation pedigree elicited in a genetics evaluation for the proband M1 (arrow) with suspected MSAc. M1’s sister was diagnosed with PD (P1) and also followed in our clinic. There were six additional relatives with PD and/or dementia. **B** Summary table of the clinical evaluation and additional tests for patients M1 and P1. Abbreviations: UMSARS: Unified MSA Rating Scale; BARS: Brief Ataxia Rating Scale; UPDRS: Unified Parkinson’s disease Rating Scale; UPSIT: University of Pennsylvania Smell Identification Test; 3D vol. MRI: 3D volumetric magnetic resonance imaging; PBRO6-TSPO-PET: translocator protein positron emission tomography; pα-syn: phosphorylated alpha-synuclein; α-syn CNVs: alpha-synuclein copy number variations. **C** 3D volumetric MRI reveals striatal, pontine, and cerebellar atrophy in M1 compared to P1. Segmentation utilized FreeSurfer software. Key structures from left to right; A: amygdala, Cc: cerebral cortex, P: putamen, Gp: globus pallidus, T: thalamus, WM: cerebral white matter, C: caudate, H: hippocampus, M: midbrain, Po: pons, Me: medulla, Cb-wm: cerebellar white matter, Cb-c: cerebellar cortex, 3: 3rd ventricle, 4: 4th ventricle, L: lateral ventricle

**Fig. 5:**
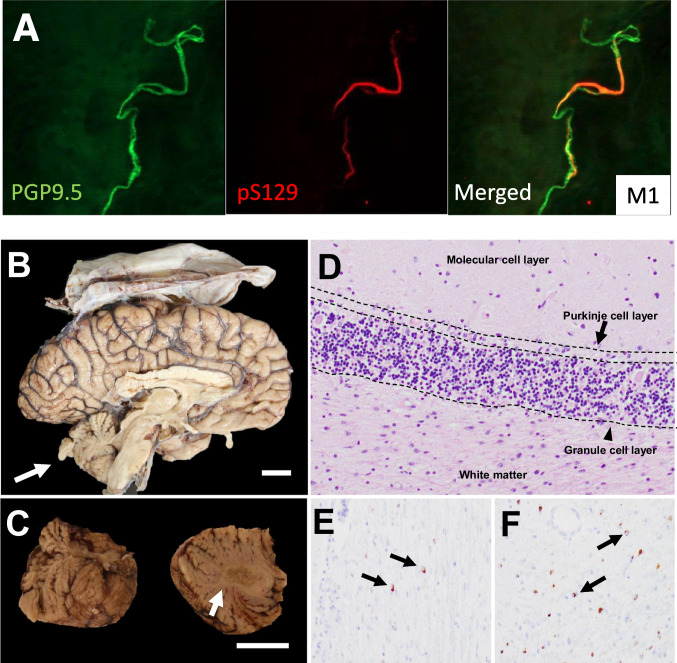
Histopathology of patient M1. **A** Immunohistochemistry of skin biopsies from M1 revealed the presence of a-synuclein phosphorylated at residue serine-129 (pS129; red) in autonomic nerve fibers positive for PGP9.5 (green). There was positive staining in patient P1 also (see Fig. 4). Abbreviations: pS129: phosphorylated a-synuclein at Serine 129, PGP9.5: protein gene product 9.5. **B**–**F** Postmortem neuropathological findings in patient M1. **B**–**C** Gross pathology shows striking cerebellar and pontine atrophy (white arrows). **D** Hematoxylin and eosin staining of the cerebellum reveals widespread loss of Purkinje cells (black arrow). **E**, **F** Immunohistochemistry for a-synuclein demonstrates the presence of glial cytoplasmic inclusions in cerebellar white matter (**D**) and pons (**E**) (black arrows). Scale bar 2 cm

**Fig. 6:**
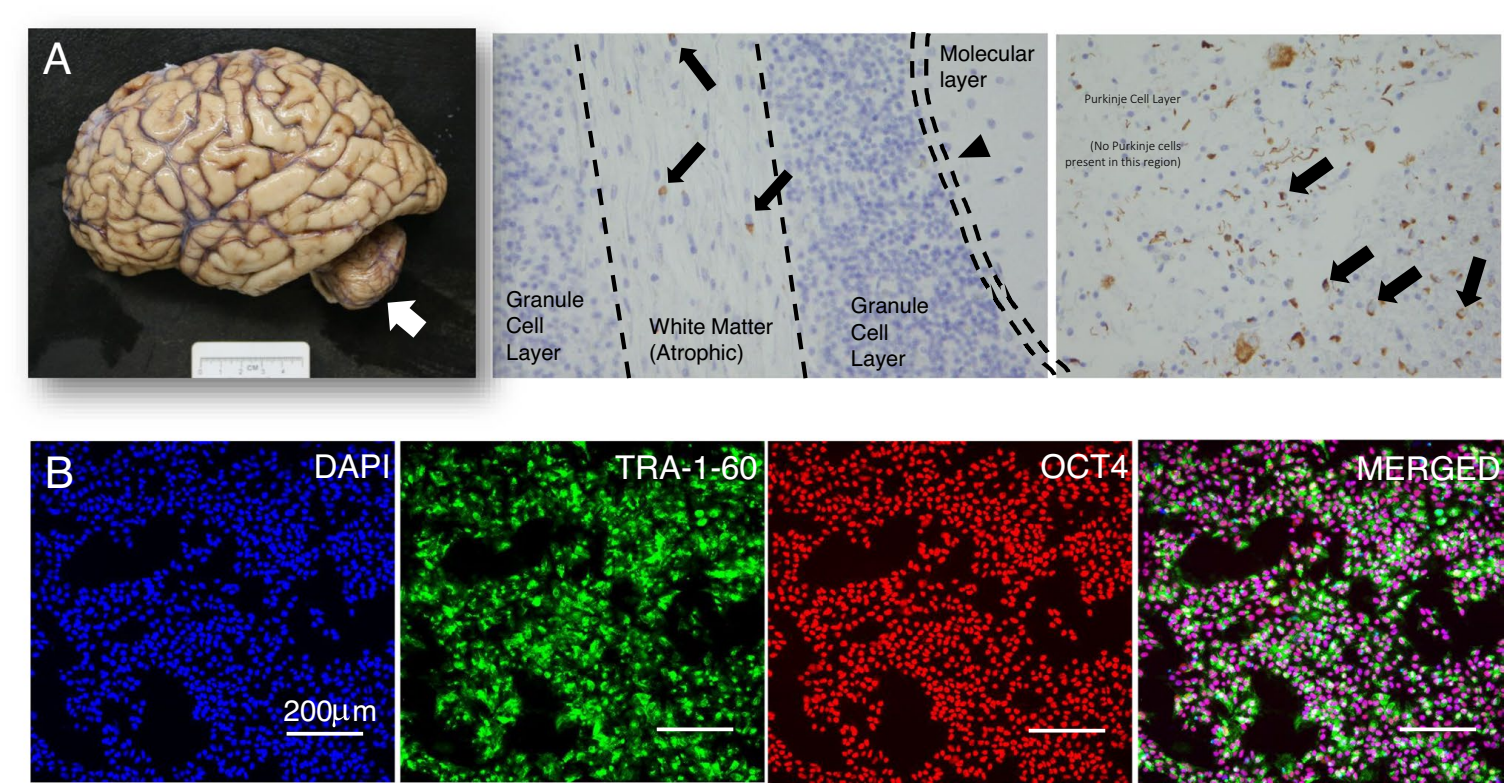
Matching brain-to-iPSc set from patient M2 (MSAc). **A** Neuropathological findings in M2 include cerebellar atrophy (left, white arrow), loss of Purkinje neurons (middle, black arrowhead), and the presence of glial cytoplasmic inclusions (black arrows) in cerebellar white matter (middle) and pons (right), as well as neuronal inclusions in pons (right). **B** iPSCs generated from M2 stain for pluripotency markers TRA-1–60 and OCT4

In Figs. 3, 4, 5, 6 we provide a case study that reflects our diagnostic workup. The proband was a 55-year-old woman (M1) initially evaluated for progressive gait and balance difficulties. During the next 7 years, the patient additionally experienced chronic urinary tract infections with urinary urgency, worsening constipation, cramping intermittent leg pain, and depression. Unusually for MSA, she had a striking family history of neurologic syndromes (Fig. 4). Her sister was diagnosed with PD, and five second-degree relatives were affected by Parkinson’s disease and/or dementia. She underwent clinical assessment, 3D volumetric MRI, UPSIT, skin biopsy (alpha-synuclein, nerve fiber density), and genetic testing. Her sister (P1 in the pedigree, Fig. 4A) underwent testing, including clinical assessment, 3D volumetric MRI, UPSIT, skin biopsy, and genetics evaluation. These data are summarized in Figs. 4 and 5. Notable findings include marked anosmia in the sister with suspected PD, and relative preservation in the proband, despite greater disease severity. Consistent with clinical diagnoses, the skin biopsy showed alpha-synuclein deposits in both patients’ cutaneous autonomic nerves (shown for M1 in Fig. 5A and not shown for P1), but decreased nerve-fiber density was noted only in the sister with PD (Fig. 4B). Figure 4C shows striatal, cerebellar, and pontine volume loss in M1 with respect to P1, also consistent with clinical suspicion. Nine years after the presentation, the proband deteriorated and transitioned to hospice. She died shortly thereafter and underwent a brain-limited autopsy, at which time a skin biopsy was also taken for iPSC generation. The autopsy confirmed the clinical suspicion of MSAc (Fig. 5B). Genetic testing has thus far been negative for SCA2, SCA3, or alpha-synuclein CNVs. Whole-genome sequencing was performed and is currently being analyzed through the Harvard Medical School Clinical Genome Analysis Platform (CGAP) and the MSA Coalition Collaborative Core G (see Discussion).

### Longitudinal Clinical Trial Paradigm: Observational to Treatment Phase Recruitment

Of our 127 patients with clinical possible/probable MSA, 69 “active” patients have been followed since 2019, when we developed our concept of a well-stratified and longitudinally tracked clinical-trial cohort. These patients were subjected to clinical investigations according to appropriate scenarios (Table 1). For more recently added testing (like MIBG scanning) it is still ongoing. These patients enter from the community from prodromal through to advanced stages. In keeping with the aggressiveness of the disease, 11 patients have died in the last 2 years. Of the 69 patients, 68 were alive in March 2020, when we began recruiting into observational studies with a view to transitioning to a longitudinal clinical trial paradigm (Figs. 1–2).

Our observational paradigm currently entails clinical assessment, MRI, neuroinflammation monitoring, biometrics, and biomarkers. Protocols are detailed in “Methods.” Briefly, clinical assessment comprises serial clinical evaluation, including three formal rating scales, Unified MSA rating scale (UMSARS [79]), Brief Ataxia Rating Scale (BARS [80]), and Montreal Cognitive Assessment (MoCA [81]). Recognizing the intrinsic and inter-rater variability limitations and crudeness of clinical assessments [82], from 2022, we will add in-person and remote biometric assessments to our longitudinal clinical paradigm. In-person assessments comprise oculomotor, speech, limb motor, balance, and cognitive tasks within a physical setup known as Neurobooth. At-home tasks comprise wearable sensors and web-based computer tasks (see “Methods”).

Our longitudinal 3 T MRI protocol includes 3D MPRAGE T1WI from which brain volumes can be calculated, as described in “Methods” and the accompanying submission [32]. Diffusion tensor imaging (DTI) and diffusion-weighted imaging (DWI) sequences are also obtained in our protocol. In addition, we monitored neuroinflammation with a PET ligand directed to the 18 kDa translocator protein (TSPO). TSPO is expressed in microglia and astrocytes [56] and the marker tracks well with the distribution of neuropathology in MSA [33]. Moreover, in familial parkinsonism, where at-risk patients are identifiable, emerging studies indicate that TSPO signal may be increased prior to motor symptoms [83]. We employ an investigational ligand [18F]PBR06 [84] and find that, consistent with other ligands, enhanced uptake tracks very well with the expected distribution of neuropathology in our patients (Fig. 3).

In addition to clinical rating scales, biometrics, and imaging, observational subjects are enrolled in three established longitudinal biobanking protocols at BWH. The first is the Harvard Biomarker Study (HBS). HBS has enrolled 3374 subjects to date, including patients with diverse neurodegenerative diseases and healthy controls. Aside from detailed longitudinal clinical assessments, the study draws blood and cerebrospinal fluid (CSF) downstream analysis (see “Methods”). CSF is also preserved for downstream analysis of patient-specific alpha-synuclein conformers through alpha-synuclein seed amplification [37]. The second is a study centered on multiple sclerosis (MS) known as the Comprehensive Longitudinal Investigations of Multiple Sclerosis at the Brigham and Women’s Hospital (CLIMB). CLIMB has enrolled 1000 patients to date, collects both blood and stool, and is well suited to study the role of neuroinflammation and the gut microbiome in MSA subjects. Finally, we recruit subjects into MGB-SCiN, a protocol in which skin biopsies at Massachusetts General Hospital or Brigham and Women’s hospital are procured for multiple tasks, including propagation of fibroblasts for iPSC production (see below), immunohistochemistry, cutaneous nerve-fiber quantitation (through contracted clinical vendor CND Life Sciences, see “Methods”), electron microscopy, and α-synuclein seed amplification [85].

### Recruitment into an Investigator-Initiated “n-of-Few” Longitudinal Clinical Trial

We recently tested how rapidly we could recruit from our active MSA cohort into a clinical intervention trial. Despite complexity introduced by the SARS-CoV-2 pandemic, we found that successful recruitment was achievable, even for an exceptionally rare disease like MSA. In March 2020, an investigator-initiated clinical intervention trial was commenced. Four patients, who already had antecedent clinical and imaging (MRI, PET-TSPO) studies, were immediately recruited. Following an extended 9-month shutdown necessitated by the SARS-CoV-2 pandemic, an additional 16 subjects were recruited over a period of 10 months from December 2020 to September 2021.

The study design comprised an antecedent observational phase, 6 months of drug and terminal clinical and imaging assessments at the conclusion of drug administration. The 4 patients who had already completed sufficient (> 6 months) observational phase underwent an additional MRI, PET-TSPO scan, and clinical evaluation, and were transitioned to 6 months of drug intervention. Of these patients, 4 of 4 completed a treatment phase and received final MRI and PET-TSPO scans. Of the 16 additional patients recruited, 7 have so far completed 6 months of drug administration. Notably, 7 patients were deemed to be too advanced (non-ambulatory) or too rapidly progressive for observational phase enrolment (from ambulatory to non-ambulatory in < 1 year or a reduction by ≥ 1 point on the UMSARS Part IV Global Disability Scale over a period of ≤ 6 months). These patients were given immediate access to the drug after a single pre-drug MRI and PET-TSPO scans, and were re-scanned after the treatment.

### Brain Banking Enrolment

At our institution, we identify patients who have a clinical diagnosis of a neurodegenerative disease. We thoroughly educate the patient and their family as far before death as possible about the utility of brain donation. An information packet and detailed protocol are provided. The patient’s healthcare proxy is notified. Official consent for brain donation and skin biopsy is obtained from a health care proxy after death. There is close communication with the funeral director, transport services, and the neuropathology team. Moreover, the availability of a pathology “rapid” team that operates outside of regular business hours helps shorten the postmortem interval (PMI). We aim for a postmortem interval of less than 4 h.

Using this approach, we have, in the past 5 years, stored 37 brains in the P + A + MSA clinic brain bank from MSA cases and related diseases including familial PD, DLB, SCA, and PSP cases. Table 2 provides a detailed list according to disease category. We fix one hemisphere in formalin for neuropathological examination (Fig. 6A) and immunohistochemistry, and flash-freeze the other half for assays including single-cell RNA sequencing, immunofluorescence, and autoradiography. In addition, to develop genotype-to-phenotype correlations, we have performed whole-genome sequencing on this set of MSA brains and related disease controls including spinocerebellar ataxias. For more details, see “Methods.”

**Table 2:** Post-mortem brain collection and hFib/iPSC lines from MSA and related disorders in the P + A + MSA brain bank

	Post-mortem brain		hFib/iPSCs (matched with p.m. brain)	
MSA	MSAc	13	MSAc 15	(11)
	MSAp	5	MSAp 5	(4)
	MSAc/p.	3	MSAc/p	2 (0)
PD	Sporadic	0	Sporadic 1	(0)
	SNCA-A53T	1	SNCA-A53T 1	(0)
	SNCA duplication	1	SNCA duplication	2 (1)
			SNCA triplication	2 (0)
DLB	Sporadic	5	Sporadic	2 (1)
	GBA	2	GBA	0 (0)
SCA	SCA-3	2	SCA-2	2 (0)
	SCA-7	1	SCA-3	2 (2)
	SCA-8	1	SCA-7	1 (1)
			SCA-8	1 (0)
PSP	PSP	1	PSP	1 (1)
	PSP-FTD	1	PSP-FTD	0 (0)
	PSPc	1	PSPc	1 (1)

Abbreviations: *hFib*, human fibroblasts; *iPSCs*, induced pluripotent stem cells; *MSAc*, cerebellar variant multiple system atrophy; *MSAp*, Parkinson-variant multiple system atrophy; *P* + *A* + *MSA*, Parkinson’s plus, ataxia, and multiple system atrophy clinic; *PD*, Parkinson’s disease; *SNCA*, a-synuclein gene; *DLB*, dementia with Lewy bodies; *GBA*, glucocerebrosidase gene; *SCA*, spinocerebellar ataxia; *PSP*, progressive supranuclear palsy; *FTD*, frontotemporal dementia; *PSPc*, cerebellar variant-PSP

### Matched Biobank-to-iPSC Program

For our matched biobank-to-iPSC program, we collect skin biopsies for fibroblast generation 5 cm above the lateral malleolus upon thorough disinfection. Ensuing fibroblast cultures are routinely tested for mycoplasma infections and propagated and frozen in large stocks. iPSCs are generated externally at Cellular Reprogramming Inc. via mRNA reprogramming (see references in the “Methods” section) and assessed for pluripotency markers (Fig. 6B). For the current study, we have generated 38 lines (Table 2) including 22 MSA lines. Familial synucleinopathy lines—including two patients with alpha-synuclein triplication, one patient with alpha-synuclein duplication, and one patient with alpha-synuclein A53T mutation—have been prioritized. As noted above, these patients can present with aggressive diffuse synucleinopathy, including MSA-like presentations with findings of oligodendroglial alpha-synuclein deposits on neuropathologic examination [9]. Of these 38 lines, 22 lines are matched to postmortem brain (15 with neuropathologically confirmed MSA). Routine quality control, including mycoplasma testing and G-band karyotyping, has been performed.

## Discussion

Here, we have outlined an approach to generate a clinical trial-ready cohort for MSA with a strategy that folds clinical trial recruitment into routine care within a clinical outpatient setting. This is practicable when formal clinical, imaging, and biometric assessments are aligned with clinic visits, especially for a patient population like MSA that can suffer from difficulties in transportation and clinical access. The combination of deep clinical phenotyping, imaging, and biobanking, including stem-cell generation from patients, may help address the need for more accurate patient stratification. Moreover, while this manuscript was in revision, the third consensus criteria for the diagnosis of MSA have been published [54]. The diagnostic work-pursue in the P + A + MSA clinic aligns very closely with these updated consensus criteria.

Beyond the diagnostic tests we specify (Fig. 2), very recent reports suggest that a brain alpha-synuclein radiotracer [41, 42, 86] may facilitate diagnosis of MSA. This would be a welcome addition to the workup, especially in conjunction with seed amplification assays (SAA) [37, 38]. Intriguingly, a recent study found that the ability or inability to amplify amyloidogenic alpha-synuclein conformers from olfactory mucosa with SAA differentiates probable MSAp from MSAc, respectively [87]. These alpha-synuclein amplification assays, whether from skin or CSF or other sources, could aid in early diagnosis, tracking disease progression, and monitoring target engagement. These will be especially pertinent for alpha-synuclein-directed therapeutics (antisense oligonucleotides, monoclonal antibodies, and small molecules) that are actively in development or already in clinical trials.

Our first attempt at recruitment of an active clinical-trial-ready cohort offers some lessons. Of the 4 patients who were actively being tracked in an early observational phase study, 4 from 4 were rapidly recruited into the investigator-initiated study and 4 from 4 of the patients completed the drug treatment. Sixty-four other patients had been diagnostically worked up by that time, but observational studies had not commenced. Of these, 16 additional patients were recruited within 9 months, despite all the challenges of a global pandemic. These numbers speak to the utility of keeping an active registry of patients and performing observational tracking in a “rolling” paradigm in between designated clinical trials. Some tests were exceptionally difficult during the pandemic, including a lumbar puncture. Now that our infrastructure is established, our expectation is that recruitment will be far smoother, and we expect our registered patients to grow in number. It will be important to find an appropriate mechanism to stratify them in the future according to severity. For some preventative interventions, a prodromal phase patient may be suitable. For other studies, a more advanced disease stage may be more appropriate. The longitudinal tracking of multiple clinical and imaging phenotypes ensures that, upon entry into the treatment phase, there is some antecedent measurable progression that can be demonstrably slowed by the therapeutic intervention.

For some investigations, notably those involving radiotracer, acceptable limits of exposure must be factored into a “rolling” longitudinal paradigm with maximum numbers of tests agreed upon through expert safety review. At a minimum, a baseline scan, a scan at the beginning of the drug intervention, and a scan at the end of the intervention are required. Also, in a longitudinal within-case paradigm (Fig. 1B), cycling of placebo and treatment may be important to determine whether a drug intervention is not only engaging its target but also responsible for any slowing of phenotypic progression. The interval of cycling will depend on the nature of the proposed therapeutic and its washout period. Six-monthly intervals (our initial paradigm) seem biologically reasonable when coupled to more sensitive measures of disease progression including emerging biomarkers and biometric assessment of movement. In the future, we will consider a 6-month placebo phase following treatment. Practicalities may make a cycling protocol with extended treatment/placebo intervals difficult for a relentless and debilitating chronic disease like MSA and may be an unattractive option for patients. For example, when we began enrolling in our observational phase, some patients, who were either non-ambulatory when referred to the clinic or exhibited rapid clinical deterioration, could not be humanely triaged into a 6-month observational phase. It remains to be seen if a cycling protocol can be effectively tried in MSA.

The clinical trial approach we allude to is in its infancy. There have been useful recent commentaries on these, including selection bias, power, trial design, and data analysis [48]. These issues will need to be addressed. In addition—and especially when there is no cycling with a placebo phase—a plausible biological mechanism is needed to explain why some patients respond or do not respond (Fig. 1B). It makes good sense to monitor as closely as possible for direct evidence of target engagement. Failure to engage a target would be the best explanation for the failure to respond to a drug. There may also be identifiable genetic factors that dictate metabolism or drug response.

In this report, we present 38 iPSC lines as a resource to the research community, including 22 lines matched to the postmortem brain. We anticipate these lines may be useful in collaborative efforts, including the MSA Collaborative Research Cores program mentioned below. Human iPSC models could prove particularly helpful in a complex disease like MSA for which a clear disease etiology and a faithful animal model have remained elusive. Already, the first studies of MSA iPSC models show promise [88–90]. In time, iPSC cultures made from our patients could enable appropriate matching of therapeutics to a patient, facilitating patient recruitment and de-risking therapeutic interventions. Recently, tremendous advances have enabled the generation of complex glio-neuronal co-cultures. 3D “mini-brain” models such as oligocorticospheroids capture all of the key CNS cell types [91]. These may be particularly pertinent to MSA, a disease that clearly involves the complex interplay of glial and neuronal cells. However, one formidable challenge in MSA iPSC modeling will be the inability to make robust mutation-corrected controls because few definitive genetic lesions have been identified. We hope that the matching of iPSC models to the postmortem brain will increase the chances that disease-relevant mechanisms are identified in our cell lines. This obstacle underscores the need for iPSC modeling to proceed in parallel with efforts in MSA genomics and environmental exposure studies.

Finally, our clinical paradigm cannot occur in a vacuum. It should occur in the context of a community of like-minded sites worldwide to capture a diverse and broad community of patients, physicians, and scientists. The MSA Coalition, a charitable organization that is a major sponsor of MSA research, is endeavoring to systematic global efforts like this. In the MSA Collaborative Research Cores initiative, like-minded groups worldwide work in close collaboration in one of four cores (G – Genetics, E – Environment, B – Biomarkers, M – preclinical Models) to address fundamental unanswered questions in MSA. These include (i) elucidation of genetic and environmental drivers of MSA risk and progression; (ii) development of better biomarkers for early diagnosis and disease activity; and (iii) establishment of better disease models to capture glioneuronal and neuroimmune interactions in MSA.

## Conclusion

It has been 7 years since the MSA Global Research Roadmap established goals through which barriers to disease-modifying MSA therapeutics may be overcome [18]. In that time, exciting advances in the field have enabled more precise clinical diagnosis of synucleinopathies. Notable advances include the identification of early and prodromal clinical features, and the development of diagnostic tests and longitudinal tracking approaches, from volumetric MRI and PET radiotracers to methods for amplifying alpha-synuclein conformers from CSF or skin. In parallel, emboldened by some stunning successes in neurogenetic disorders, there have been bold new innovations in clinical trial design, recognizing that n-of-1 or n-of-few clinical trials may be appropriate in patients with devastating neurological disorders [75, 76]. We have successfully implemented an integrative approach between the clinic and longitudinal clinical trial recruitment. We anticipate our approach will not only facilitate more seamless recruitment of MSA patients into such studies, but engender a deeper biological understanding of the nature of this complex and devastating disease.
