# Correction to: Clinical trial-ready patient cohorts for multiple system atrophy: coupling biospecimen and iPSC banking to longitudinal deep-phenotyping

**DOI:** 10.1007/s12311-022-01501-5

**Published:** 2022-12-02

**Authors:** Alain Ndayisaba, Ariana T. Pitaro, Ashlan S. Willett, Kristie A. Jones, Claudio Melo de Gusmao, Abby L. Olsen, Jisoo Kim, Eero Rissanen, Jared K. Woods, Sharan R. Srinivasan, Anna Nagy, Amanda Nagy, Merlyne Mesidor, Steven Cicero, Viharkumar Patel, Derek H. Oakley, Idil Tuncali, Katherine Taglieri-Noble, Emily C. Clark, Jordan Paulson, Richard C. Krolewski, Gary P. Ho, Albert Y. Hung, Anne-Marie Wills, Michael T. Hayes, Jason P. Macmore, Luigi Warren, Pamela G. Bower, Carol B. Langer, Lawrence R. Kellerman, Christopher W. Humphreys, Bonnie I. Glanz, Elodi J. Dielubanza, Matthew P. Frosch, Roy L. Freeman, Christopher H. Gibbons, Nadia Stefanova, Tanuja Chitnis, Howard L. Weiner, Clemens R. Scherzer, Sonja W. Scholz, Dana Vuzman, Laura M. Cox, Gregor Wenning, Jeremy D. Schmahmann, Anoopum S. Gupta, Peter Novak, Geoffrey S. Young, Mel B. Feany, Tarun Singhal, Vikram Khurana

**Affiliations:** 1 Department of Neurology, Building for Transformative Medicine Room 10016L, Brigham and Women’s Hospital and Harvard Medical School, 60 Fenwood Road, Boston, 02115 USA; 2 Division of Clinical Neurobiology, Department of Neurology, Medical University of Innsbruck, Anichstraße 35, 6020 Innsbruck, Austria; 3 Department of Radiology, Brigham and Women’s Hospital and Harvard Medical School, Boston, MA 02115 USA; 4 Department of Pathology, Brigham and Women’s Hospital and Harvard Medical School, Boston, MA 02115 USA; 5 Present Address: Department of Neurology, University of Michigan, Ann Arbo, MI 48103 USA; 6 Department of Pathology, Massachusetts General Hospital and Harvard Medical School, Boston, MA 02114 USA; 7 Department of Neurology, Massachusetts General Hospital and Harvard Medical School, Boston, MA 02114 USA; 8 Cellular Reprogramming, Inc, Pasadena, CA USA; 9 The Multiple System Atrophy Coalition, Inc, 7918 Jones Branch Drive, Suite 300, McLean, VA 22102 USA; 10 Department of Pulmonary, Sleep and Critical Care Medicine, Salem Hospital, MassGeneral Brigham, Salem, MA 01970 USA; 11 Department of Urology, Brigham and Women’s Hospital and Harvard Medical School, Boston, MA 02115 USA; 12 Department of Neurology, Beth Israel Deaconess Medical Center and Harvard Medical School, Boston, MA 02115 USA; 13 Laboratory of Neurogenetics, Disorders and Stroke, National Institute of Neurological, National Institute of Neurological Disorders and Stroke, Bethesda, MD 20892 USA; 14 Department of Neurology, Johns Hopkins University Medical Center, Baltimore, MD 21287 USA; 15 Department of Biomedical Informatics, Harvard Medical School, Boston, MA USA; 16 Division of Genetics, Department of Medicine, Brigham and Women’s Hospital, Boston, MA 02115 USA

**Correction to: The Cerebellum**

**https://doi.org/10.1007/s12311-022-01471-8**

In the course of preparation of the document, an author with a critical contribution, Dr. Anoopum Gupta, was dropped from the author list inadvertently due to a clerical error.

Dr. Gupta contributed the Neurobooth methodology to this work. We have now amended the author list and also the Funding section as presented below.

## Data Availability

None.

